# Mammalian Sterile20-like Kinases: Signalings and Roles in Central Nervous System

**DOI:** 10.14336/AD.2017.0702

**Published:** 2018-06-01

**Authors:** Sheng Chen, Yuanjian Fang, Shenbin Xu, Cesar Reis, Jianmin Zhang

**Affiliations:** ^1^Department of Neurosurgery, The Second Affiliated Hospital, School of Medicine, Zhejiang University, Hangzhou, Zhejiang, China.; ^2^Department of Physiology and Pharmacology, Loma Linda University, Loma Linda, California, USA.; ^3^Brain Research Institute, Zhejiang University, Hangzhou, Zhejiang, China.; ^4^Collaborative Innovation Center for Brain Science, Zhejiang University, Hangzhou, Zhejiang, China.

**Keywords:** Mammalian Sterile20-like kinases, central nervous system disorders, mitogen-activated protein kinase

## Abstract

Mammalian Sterile20-like (MST) kinases are located upstream in the mitogen-activated protein kinase pathway, and play an important role in cell proliferation, differentiation, renewal, polarization and migration. Generally, five MST kinases exist in mammalian signal transduction pathways, including MST1, MST2, MST3, MST4 and YSK1. The central nervous system (CNS) is a sophisticated entity that takes charge of information reception, integration and response. Recently, accumulating evidence proposes that MST kinases are critical in the development of disease in different systems involving the CNS. In this review, we summarized the signal transduction pathways and interacting proteins of MST kinases. The potential biological function of each MST kinase and the commonly reported MST-related diseases in the neural system are also reviewed. Further investigation of MST kinases and their interaction with CNS diseases would provide the medical community with new therapeutic targets for human diseases.

Mitogen-activated protein kinases (MAPKs) signal cascades are highly conserved across eukaryotes and have a pivotal role in meditating cell proliferation, cell differentiation, stress-related response and cell death [1]. These cascades consist of three-tiered kinases (MAPKs, MAPK kinases and MAPK kinase kinases), which transduce developmental and environmental stimuli signals to the target proteins [2]. Sterile 20 (STE20) kinases are located upstream in the MAPK pathway in budding yeast, subsequently invoking the MAPK kinases to initiate the MAPKs [3, 4]. According to their distinct structures and functions, STE20 kinases can be broadly divided into two subfamilies, p21Rac/Cdc42-activated kinases (PAKs) [5] and germinal center kinases (GCKs) [6]. As the mammalian homologues of STE20, Mammalian Sterile20-like (MST) kinases considered as a part of the GCK subfamily [7]. Research demonstrated that MST kinases play an essential role in responding to control of organogenesis, organ size, and immune formation by regulating cell proliferation, differentiation, renewal, polarization, and migration [8-11].

The central nervous system (CNS) is a sophisticated entity, with two parts including brain and spinal cord. CNS takes charge of information reception, integration, and responses derived from messages provided by the many organ systems of the body. Several pathophysiological mechanisms participate in development of CNS diseases and injuries, such as neural apoptotic and necrosis, oxidative damage, inflammation, ischemia, demyelination, excitotoxicity and injuries of astrocytes, oligodendrocytes, and axonal and genetic disorders [12-14]. These alterations in CNS would normally result in sensory, motor and cognitive dysfunction or even death. Spontaneous regeneration of neurons is not possible, leaving few treatment options for patients with CNS injuries or diseases [15, 16].

**Figure 1.**
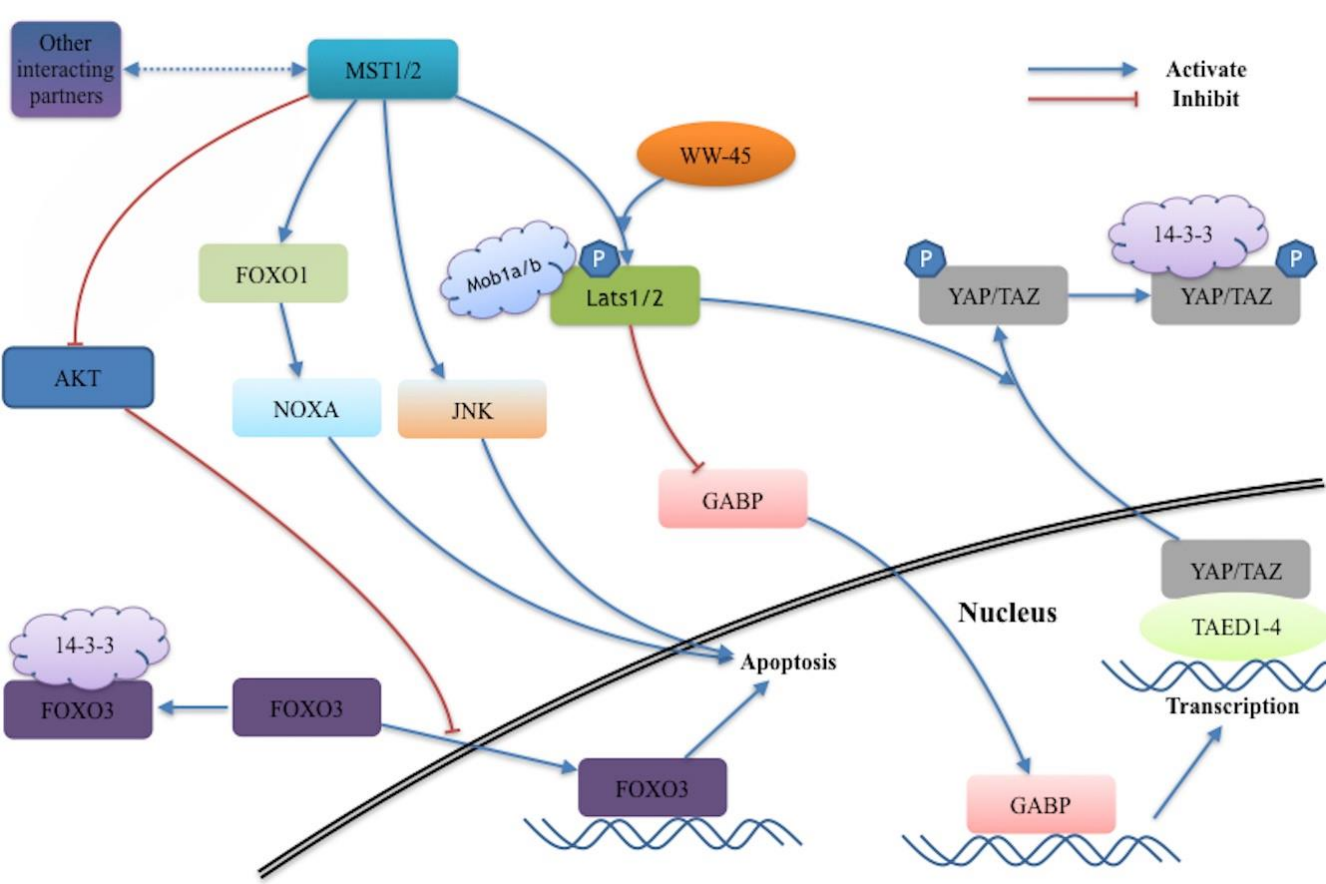
Signaling network of MST1 and MST2 kinase. The signaling pathways of MST1/2 mainly include MST1/2-YAP/TAZ signaling pathway and MST1/2-FOXO signaling pathway. MST1/2 phosphorylates the downstream Lats1/2 and subsequently inhibits the transcriptional function of intranuclear YAP/TAZ, avoiding the excessive cell proliferation and organ overgrowth. The MST1/2 can also mediate this signaling pathway by suppressing GABA function. The MST1/2-FOXO signaling pathway mainly regulates the apoptosis process. MST1/2 phosphorylates AKT and subsequently disrupts its function of interaction between FOXO3 with 14-3-3 proteins. This indirectly promotes apoptosis process. In addition, Mst1 promotes the proapoptotic mediator NOXA transcription via the assistance of FOXO1. The MST1-induced JNK activation can also promote apoptosis. Several interacting partners such as RASSF members, DAP4, acinus and Raf-1 also combine with the MST1/2 and perform different biological functions.

Given the potent effects of MST signaling networks on different biological functions, MSTs are evolving in the development of several diseases including cancer[17], endothelial pathologies[18], autoimmunity[19] and organ dysplasia[20, 21]. Recently, accumulating evidences have demonstrated how MST kinases play important roles in neuronal signal pathway and development of CNS diseases[22-25]. Therefore, a circumstantial recognition of the roles MST kinases play in the CNS would significantly broaden the horizon on potential targets for therapy to treat neuronal diseases. This review focuses on the biological function and underlying mechanism of MST kinases in the pathogenesis of diseases in CNS.

## Signaling Network and Biological Functions of MST Kinases

**Figure 2.**
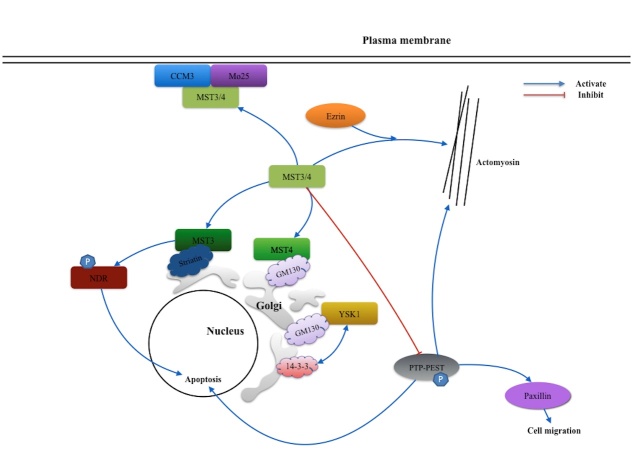
Signaling network of MST3, MST4 and YSK1 kinase. MST3, MST4 and YSK1 located on the Golgi apparatus with the assistance of GM130 and Striatin proteins. Unlike MST3 and MST4, YSK1 acts a positive role when localized to the Golgi via interaction with 14-3-3. This link potentially mediates the protein transport, cell polarity and cell adhesion. CCM3 or Mo25 induces the movement of MST3 and MST4 from the Golgi apparatus to the plasma membrane. Activated MST3/4 can promote co-localization of the actomyosin with help of Ezrin. Besides, MST3 inhibit PTP-PEST and prevent PTP-PEST dependent paxillin phosphorylation which consequently attenuates the cell migration. MST3 also can activate the NDR protein kinases to regulate the apoptosis process.

In general, there are five MST kinases including MST1 or serine/threonine-protein kinase 4 (STK4), MST2 (STK3), MST3 (STK24), MST4 (STK26), and YSK1 (STK25) that exist in mammalian signal transduction pathways [26]. MST1 and MST2 (Hippo pathway in Drosophila) are considered as the members of germinal center kinase (GCK) II subfamily. The MST3, MST4, and YSK1 are divided into the GCKIII subfamily [7, 27]. With assistance from the sequence analysis and function data, it was accepted that the yeast kinases Cdc15 and Sid1 are most matched to MST1 and MST2, while Kic1 and Nak1 in yeast are most matched to the MST3, MST4, and YSK1 [26]. The biological functions of these kinases include their roles in signal transduction pathways which help maintain cell homeostasis. Here we introduced these kinases in two parts: MST1 and MST2 (Fig. 1); MST3, MST4 and YSK1 (Fig. 2).

## MST1 and MST2

With the assistance of cofactor WW-domain scaffolding protein WW45 (Sav in Drosophila), MST1/2 binds and phosphorylates the downstream Nuclear Dbf2-related (NDR) family kinases and Large tumor suppressor (Lats1/2, as Warts in Drosophila), through interaction with SARAH domains [28]. Meanwhile, phosphorylation of Mps one binder kinase activator-like 1 (Mob1, Mats in Drosophila) a/b by MST1/2 promotes the integration between Mob1a/b and Lats1/2 and actives the Lats1/2 [29]. With the induction of Lats1/2, two intranuclear transcriptional regulators, Yes-associated protein (YAP) and Transcriptional coactivator with PDZ-binding motif (TAZ), which are similar as Yki in Drosophila, interact with 14-3-3 proteins and subsequently lose their function in cytoplasm [30]. This mechanism negatively attenuates transcription activity from losing interaction between YAP/TAZ and their target genes. Intranuclear YAP interacts with the TEAD1-4 transcription factor to stimulate cell proliferation and against the cellular death [31]. Thus, the mammalian Hippo pathway avoids excessive proliferation and organ overgrowth in stem cells [31, 32]. A recent study found that MST1 and MST2 signal also inhibits YAP function by suppressing GA-binding protein (GABP) transcriptional activity [33]. Additionally, the Hippo pathway can regulate polarization of the F-actin cytoskeleton in epithelial cells and migrating border cells [34, 35]. This mechanism was demonstrated in the Drosophila through inhibiting the Ena/Capping protein system instead of Yki [34, 35].

MST1 and MST2 kinase can also activating phosphorylation of the forkhead box proteins (FOXO) and subsequently promote the proapoptotic genes expression [7, 36]. MST-FOXO pathway is mainly initiated under the circumstances of stress. It regulates cell death by means of phosphorylating FOXO1 at serine 212 or the FOXO3 at serine 207 in neurons [22, 36]. Meanwhile, MST1 and MST2 can also phosphorylate the survival kinase AKT and inhibit its activation on FOXO3, indirectly disrupting its interaction with 14-3-3 proteins and promoting the apoptosis [36, 37]. MST1 promotes the proapoptotic mediator NOXA transcription via the assistance of FOXO1. This signaling pathway mainly works in controlling apoptosis in cancer cells [37]. Additionally, recent finding show how MST1/2 phosphorylates and stabilizes the transcription factor forkhead box A2 (FOXA2), which plays an essential role in the regulation of pneumocyte maturation and surfactant homeostasis [38]. Lastly, the MST1-FOXO signaling pathway is beneficial for the immune system in maintaining naive T cell homeostasis [39].

**Figure 3.**
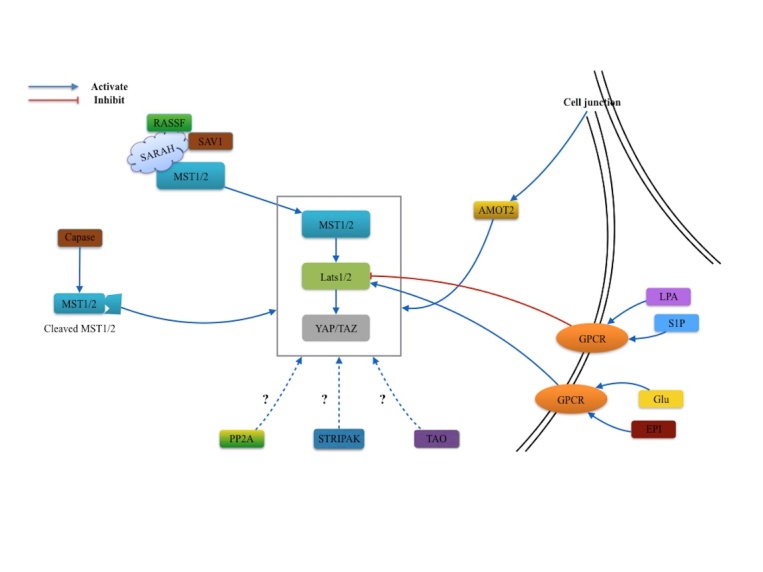
Regulators of MST1 and MST2. Several regulators are involved in the MST1/2 signaling pathway. AMOT2 protein may potentially activate the Hippo-YAP pathway by sensing the mechanical alteration from the cell-cell junction. With the assistance GPCR, biological activation mediate Lats1/2 function in different circumstances. In addition, the SAV1/WW45 and RASSF can activate the MST1/2 through the interaction with SARAH domain. Caspase proteins from apoptotic processes can cleave the MST1/2 and promote human Hippo pathway. While PP2A, STRIPAK complex and TAO may own the potential effect on this pathway.

Another downstream protein of MST1, JNK, is activated under the induction of discharged or overexpressed MST1, which was shown to be important for chromatin condensation during apoptosis[40]. A previous study proved the MST1 and MST2 can activate the JNK-SAPK pathway to induce cell cycle arrest, apoptosis, or cell survival in response to the actin cytoskeleton disruption [41]. Some studies show that MST1 and MST2 have several interacting partners, including RASSF members, DAP4, acinus and Raf-1. They combine with the MST1 and MST2 and promote different biological functions in cellular signal pathway [7].

**Figure 4.**
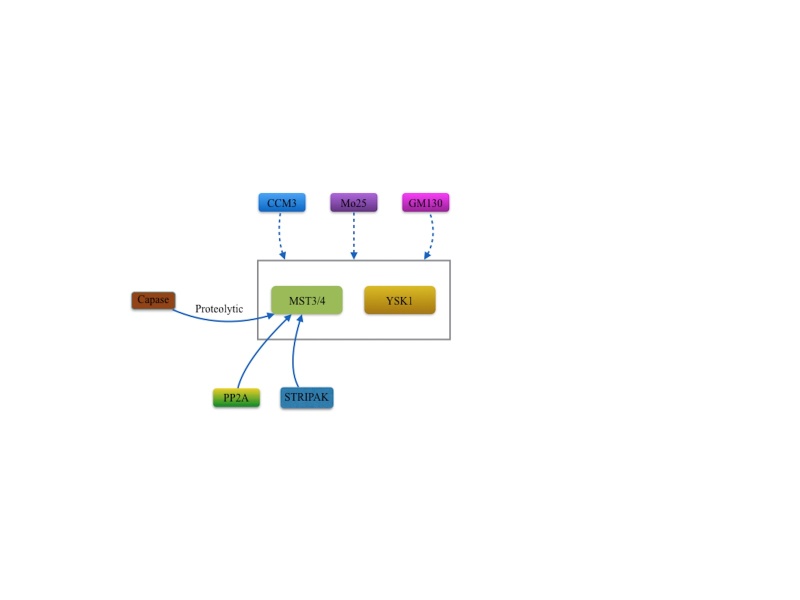
Regulators of MST3 and MST4 and YSK1. The biological function of MST3, MST4 and YSK1 kinases mainly depend on the interaction with GM130, Mo25 and CCM3 binding proteins. Caspase proteins from apoptosis, PP2A and STRIPAK complex also can regulate MST3 or MST4.

## MST3, MST4 and YSK1

Compared to the MST1 and MST2 kinases, less is known about MST3, MST4, and YSK1 kinases. The MST3, MST4, and YSK1 kinases play essential roles in regulation of cell polarity, cell adhesion, and cell migration. Normally, with assistance of different Golgi matrix proteins, these three kinases are recruited to the Golgi apparatus [11, 42, 43]. GM130 may be the common protein of MST4 and YSK1 that help target the Golgi apparatus. MST3 localize on the Golgi through interaction with Striatin proteins [11, 43, 44], and this interactions inactives MST3 and MST4. In contrast, YSK1 binding the GM130 may play a positive role on Golgi apparatus. The biochemical screening identifies YSK1 as locating to the Golgi apparatus via a special substrate, 14-3-3 proteins. This link causes potential concern for protein transport, cell polarity, and cell adhesion[11]. The adaptor proteins, cerebral cavernous malformation 3 (CCM3) protein or Mo25 can induce the movement of MST3 and MST4 from the Golgi apparatus to the plasma membrane. Active MST3 and MST4 promote the co-localization of the actomyosin with the help of Ezrin protein [26, 45]. In addition, MST3 directly phosphorylates the downstream protein-tyrosine phosphatase (PTP)-PEST and inhibits the activity of tyrosine phosphatase activity on PTP-PEST. This reaction inhibits the PTP-PEST dependent paxillin phosphorylation and consequently attenuates cell migration [42]. The PTP-PEST is involved in actin cytoskeleton regulation and actively contributes to the cellular apoptotic response [46]. As in the pathway of MST1 and MST2 on Lats, MST3 can phosphorylate and activate the NDR protein kinases at the site of Thr442/Thr444. It also regulates cell cycle progression and morphology control [47]. However, evidence of MST4 or YSK1 directly phosphorylating NDR protein kinases in mammalian systems remains unclear [48].

## Regulation of MST kinases

Regulation of MST kinases is complicated and the current studies on regulatory mechanisms are not well understood. Here we conclude with several potential regulator signals of MST kinases (Fig. 3 and Fig. 4)

MST1 and MST2 kinase play important roles in cell proliferation and tissue size via the MST1/2-YAP/TAZ signal pathway. However, the upstream regulator of this pathway remains unclear. The angiomotin 2 (AMOT2) protein may be the potential activator of Hippo-YAP pathway by sensing the mechanical alteration from the cell-cell junction [49, 50]. The G-protein-coupled receptor (GPCR) signaling modulates the Hippo-YAP pathway through controlling Lats1/2 activity. In abnormal conditions, serum-borne lysophosphatidic acid (LPA) and sphingosine 1-phosphophate (S1P) stimulate the G12/13-coupled receptors to inhibit the Lats1/2 function. In contrast, glucagon and epinephrine induce GPCR and activate the Lats1/2 function in the homeostatic condition [51].

The MST1 and MST2 kinase can also be regulated through the SARAH domain, which promotes MST1 trans-autophosphorylation and the MST2 activation loop catalyzed within the homodimer [52]. The SARAH domain is a specialized coiled coil domain near the C-terminal of MST1 and MST2, named from the three gene families, Salvador(SAV1)/WW45, RASSF and Hippo/Mst1/Mst2 [53]. The SARAH domain binds to RASSF family proteins and SAV1,positively stimulating MST1/2 activity [54, 55]. Previous studies showed cleavage sites of MST1/2 caspases located on the distal catalytic domain can be activated during apoptosis[56-58]. The caspase cleaved ~36kDa Mst1/2 polypeptides can also initiate nuclear access of human Hippo pathway, which is less impeded than the traditional pathway. However the exact mechanism is still unknown [52].

Furthermore, Hippo activities can also be regulated by the protein phosphatase (PP) 2A, Striatin Interacting Phosphatase and Kinase (STRIPAK) complex and TAO kinases in the Drosophila studies [59, 60]. Despite the known interactive effects of these regulators with the mammalian MST1 and MST2 details remain vague and requires more investigative studies.

The regulators of MST3, MST4, and YSK1kinases include GM130, Mo25 and CCM3 binding protein, and STRIPAK. As discussed above, the MST3, MST4, and YSK1 kinases play different roles in the cellular functions by combining different adaptor proteins (GM130, Mo25 and CCM3) and their locations. One study suggested that apoptotic regulatory protein caspase are able to mediate the proteolytic activation of MST3. With the assistance of this mechanism, nuclear translocation and apoptosis were induced [61]. Suppression of PP2A activity or disruption of STRIPAK complex promotes the phosphorylation of the activation loop of MST3 and MST4 [45].

**Table 1:** Main findings of MST kinases in CNS biological function.

Author/Year	MST kinase	Signaling pathway	Main function
Lehtinenet al.[36]/2006	MST1	MST1-FOXO3	Mediates oxidative-stress-induced cell death
Yuan et al.[22]/2009	MST1	MST1-FOXO1	Mediates survival factor deprivation-induced cell death
Xiao et al.[67]/2011	MST1	c-Abl-MST1-FOXO3	Mediates oxidative-stress-induced cell death
Yun et al.[70]/2011	MST1	IFN-γ-Daxx-MST1	Mediates proinflammatory-cytokine-induced cell death
Lee et al.[68]/2014	MST1	MT3-zin-c-Abl-MST1	Mediates oxidative-stress-induced cell death
Liu et al.[25]/2012	MST2	c-Abl-MST2	Mediates oxidative-stress-induced cell death
Tang et al.[71]/2014	MST3	Cdk-5-MST3-RhoA	Mediates RhoA-dependent actin dynamics and neuronal migration
Ultanir et al.[48]/2014	MST3	MST3-TAO1/2	Promotes spine synapse development
Zhou et al.[24]/2000	MST3b	PKA-MST3b	Mediates MAPK pathways
Irwin et al.[72]/2006	MST3b	Neurotrophic-MST3b	Promotes axon outgrowth
Lorber et al.[14]/2009	MST3b	/	Promotes axon regeneration
Fidalgo et al.[74]/2012	MST4	MST4-ERM	Prevents oxidative-stress-induced cell death
Matsuki et al.[77]/2010	STK25	LKB1-STK25-GM130	Mediates Golgi dispersion, axon specification and dendrite growth
Zhang et al.[76]/2012	STK25	CCM3—STK25	Promotes oxidative-stress-induced cell apoptosis
Matsuki et al.[79]/2013	STK25	/	Acute inactivation of STK25 instead of constitutive STK25 deficiency disrupts the neuronal migration

The MST1/2 mainly mediates stress-induced cell death. And the different regulators on the upstream or downstream also was introduced in past. While MST3 or MST3b mainly promoted axon outgrowth through several signaling pathway. MST4 and YSK25 can also mediate stress-induced cell death. Besides, STK25 also can mediate neuronal migration.

## The biological function of MST kinases in central nervous system

MST kinases exist in the mammalian cellular signal transductions to control cell homeostasis and survival involving cell apoptosis, differentiation, migration, and transformation [7]. Evidence showed MST kinases have essential roles in the immune system [62, 63], digestive system [20, 64], cardiovascular system [65, 66], respiratory system [38] and CNS. We give an overview of MST kinases and their various roles in CNS function (Table 1).

MST1 and MST2 contribute critical roles in regulation of neural cell death. Under oxidative stress stimulation, MST1 is activated in mammalian neurons and catalyze phosphorylation on transcription factor, FOXO3, at serine 207 [36]. It steers the FOXO3 away from the 14-3-3 proteins and aids in its translocation to the nucleus. Accumulation of nuclear FOXO3 induces expression of cell death genes [36]. Another FOXO family protein FOXO1 can also be phosphorylated by MST1 at serine 212 in granule neurons deprived of growth factors and neuronal activity [22]. Similarly, it triggers disruption of associations between FOXO1 and 14-3-3 proteins and subsequently leads to cell death [22]. These two findings demonstrate how MST1-FOXO signaling acts as an important link in the stress-induced neuronal cell death. Protein kinase c-Abl also regulates MST1 by phosphorylating it at Y433 during oxidative stress responses of neurons [67]. A recent study showed that c-Abl situated upstream of MST1-FOXO3 signaling pathway promotes cell death processes in both rat hippocampal neurons and primary culture neurons [67]. However, another study suggested a mutual regulatory dynamic mechanism exists between Mst1 and c-Abl under the conditions of oxidative stress in astrocytes [68]. The zinc-binding protein metallothionein-3 (Mt3) contributes to the activation of c-Abl through its interaction with actin [69]. MST1 and c-Abl may be regulated by Mt3 by sensing the level of free zinc separated from zinc-Mt3complex after oxidative stress induction [68]. Recently, MST2 was found to be phosphorylated by c-Abl at Y81, an evolutionarily conserved site within the kinase domain. This reinforced the function of the c-Abl-MST signaling cascade in neuronal cell death after oxidative stress [25]. Simultaneously, after injury or infection of the CNS, elevated interferon-γ (IFN-γ) can induce expression of the Daxx in microglial cells which are located in macrophages and are first responders to outer stimulation in the brain [70]. Subsequently, the microglial cells mediate the MST1 activities, such as activation, homodimerization, and nuclear translocation. This mechanism upregulates the IFN-γ induced microglial cell death when compared with the MST1-null mice [70].

MST3, MST4, and YSK1 kinases regulate cell polarity and migration through interaction with different proteins [11, 42-44]. MST3 silencing in utero can significantly disrupt the multipolar-to-bipolar transition in the migrating neurons. The serine/threonine kinase, cyclin-dependent kinase 5 (Cdk5) was shown to be the upstream regulator of the MST3 though its phosphorylating effect at Ser79 site of MST3 [71]. Furthermore, MST3 regulated neuronal migration by phosphorylating the RhoA at Ser26 and modulating its activity with GTP, contributing to actin dynamics in neurons [71]. A recent study showed MST3 enhances the development of proper filopodia, dendritic spine, and excitatory synapse by phosphorylating the downstream effector TAO1/2, the microtubule and actin interactor binding proteins [48]. Phosphorylated TAO1/2 subsequently enables Myosin Va function to promote the development of dentridic spine synapse [48].

MST3b is a special isoform of MST3 kinase, identified and discerned from MST3 in the 5′ coding region. It is mainly expressed in brain regions [24]. The AMP-dependent protein kinase (PKA) directly phosphorylates the MST3b, but not MST3 at amino terminus Thr-18 to regulate the MAPK pathways [24]. In addition, it can be activated by neurotrophic factors and promotes axonal outgrowth in neurons, such as adult optic nerve and radial nerve [14, 72]. However, the potential mechanism that exists between MST3b and axonal outgrowth remains less well understood and requires a further investigation.

CCM3, also called PDCD10 (programmed cell death 10), is closely related to the functions of GCKIII family of protein kinases whose mutation can lead to the development of CCMs in the brain[73]. The MST4 kinase has the capacity to phosphorylate cytoskeletal proteins, including ezrin/radixin/moesin (ERM) family proteins, in response to the oxidative stress[74]. Under the normal circumstances, MST4 mainly locates on the cis-Golgi, forming a complex with GM130 in unstressed cells. However, MST4 is activated by the CCM3 after exposure to reactive oxygen species and executes a prosurvival role to face cell death [75]. Another GCKIII family member STK25 (namely YSK1) is also activated under stimulation of oxidative stress. The interaction between STK25 and CCM3 further regulates the extracellular signal-regulated kinase. This reaction aggravates the cell apoptosis [76]. STK25 overexpression promotes Golgi condensation and multiple axons with the assistance of protein kinase LKB1 in the nervous system [77]. Interestingly, the LKB1-STK25-GM130 signaling parallels with the Reelin-Dab1 signaling and presents an antagonistic phenomenon on neural polarization, morphogenesis, and Golgi distribution [77]. The Reelin-Dab1 signal pathway is responsible for the cell positioning, synaptic circuit formation, and neuronal migration during development of the CNS [78]. However, it is unknown whether STK25 affects neuronal migration and involves the Reelin pathway. The following study found that acute inactivation of the STK25 can disrupt the neuronal migration instead of constitutive STK25 deficiency. In addition, LKB1, STRAD and GM130 also take part in this process [79]. Despite the current understanding, details on how these proteins interact to regulate neuronal migration during development is a topic for future studies.

## The MST kinases in central nervous system disorders Brain Tumor

As discussed above, the MST1/2-Lats-YAP/TAZ signaling pathway has potent effects on regulating cell proliferation, and was accepted as a potential mechanism of tumor growth. The evidence shows that down-regulated MST1 expression can be a tumor growth marker of colorectal cancer or lymphoma/leukemia[80, 81]. Decreased Lats level can be observed in the breast cancers and mesotheliomas [82, 83]. While up-regulated YAP/TAZ expression has also been demonstrated taking parts in cancer development, such as lung cancer [84], hepatocellular carcinoma [85], colorectal cancer [86], pancreatic ductal[84], medullablastoma [82] and neurofibromatosis type2 [87]. Meanwhile, this phenomenon was also detected in cerebral malignant tumor at the mRNA and protein levels. Moreover, they found new evidence that YAP/TAZ can abnormally activate the target gene, BIRC5, accompanied with down-regulation of LATS1/2, in turn simulating aberrant cell growth and neoplasia in glioblastoma [88]. However, the upstream regulator of MST1/2-Lats-YAP/TAZ signaling pathway remains unknown in their study. MicroRNAs (miRNAs) are critical components in human tumorigenesis with the assistance of their mRNA 3′ untranslated regions [89, 90]. Recently, a study found that miR-130b, situated upstream of MST1/2-Lats-YAP/TAZ, was substantially overexpressed in human glioblastoma growth. Hyperactivation of miR-130b directly suppressed MST1 activity, further leading to YAP/TAZ activation [91].

The survival kinase AKT is located downstream of the phosphatidylinositol 3-kinase (PI3K), is constitutively activated and regulates human cancer and precancerosis progression [92-94]. It has been shown this mechanism also functions through signaling between AKT and downstream mammalian target rapamycin (mTOR) [95, 96]. Similarly, another study found MST1 binds to AKT and attenuated the AKT and mTOR activity in glioma cell. This finding further suggested that MST1-AKT-mTOR signaling pathway is involved in glioma cell proliferation and growth. However, YAP expression was not affected in this study [97].

Although MST3 and MST4 have rarely been introduced in tumorigenesis, they may play a positive role in tumorigenesis due to the function of cell migration. Studies found that rising MST4 and CCM3 expression level increases breast cancer progression and poor prognoses [45, 98]. While the involvement of MST3/4 in CNS tumorigenesis still needs further investigation, another GCKIII family member, STK25, was shown to mediate TrkA-CCM2 death signaling in medulloblastoma cells [99]. The TrkA regulates tumor cell death in neuroblastoma via its interaction with protein product of CCM2 [100]. Subsequent findings show that STK25 can phosphorylate CCM2 and initiate death signaling in medulloblastoma cells [99].

## Cerebrovascular Diseases

Cerebrovascular diseases include cerebral hemorrhage, ischemic, arterial or venous malformations, or other vascular lesions [101]. Death from stoke is three times more likely than death from coronary cardiovascular diseases in China [102]. The most common disease concerning MST kinases is CCM, which can change neurological function via hemorrhage, vascular steal, venous congestion, or compression effect on normal brain tissue [103]. Disorders in the signaling pathways of MST3, MST4, and STK25 are the widely accepted factor in CCM pathology [18, 104]. The CCM gene family includes CCM1, CCM2, and CCM3. The clinical manifestations of patients suffering genetic mutations of these three genes are similar, suggesting a common pathway integrated with their expression [105]. Previous studies demonstrated that STK24 and STK25 interact with CCM3, but only STK25 has the ability to phosphorylate CCM3 [75, 106]. STK25 forms a protein complex with CCM2 [106]. However, the specific mechanism and potential relationship between STK25, CCM2, and CCM3 remains vague. This link between CCM2, CCM3, and STK25 can be the essential part of signaling pathways in CCM pathogenesis. A recent study proposed that STK24 and STK25 control endothelial cell-cell junctions through directly activating ERM family protein, which negatively regulates Rho. They speculated that the CCM3/STK signaling may share a common pathway with STK/ERM/Rho signaling to regulate epithelial and endothelial cell junctions. This is essential in cardiovascular development and diseases such as CCM [104]. While it is obvious that GCKIII family proteins widely participate in endothelial pathologies, more work is required to effectively elaborate on the exact relationship between GCKIII family proteins and CCMs.

Cerebral ischemia is the main cause of strokes, accounting for 80% of cerebrovascular accidents [107]. It causes the highest rate of disability, is the second cause of dementia, and the fourth cause of death in development countries [107]. A recent study demonstrated that MST1 signaling is important for oxidative stress-induced neuronal cell death after cerebral ischemia via microglial activation [108]. Microglial activation is an important step in ischemic stroke-induced immune alteration and essential for oxidative stress-induced neuronal cell death [109, 110]. A recent study demonstrated that MST1 regulates microglial activation via directly phosphorylating substrate protein IκBα and subsequently initiating the nuclear factor-kappa B (NF-κb) activation. NF-κb is thought to delayed inflammation and neurotoxicity in microglia [108, 111]. Upstream kinase Src was was showed to regulate MST1-IκB signaling during microglial activation. This novel pathway is a potent therapeutic target for ischemic stroke[108]. Furthermore, another study found the Hopeahainanensis leaf extract oligomer compound, Malibatol A, can prevent oxygen-glucose deprivation (OGD) induced BV2 cell injury via the potential c-Abl/MST1 signaling pathway [112]. The BV2 cell is the common alternative candidate of microglia, as it is similar in morphology, phenotypes and functions [113]. The balance between M1 and M2 microglia marker of BV2 cell was modulated by Malibatol A which effectively attenuated cell injury after OGD stimulation [112]. This balance is crucial for improving neurological disease outcomes [114]. Despite enhanced expression of p-MST1 and c-Abls after OGD, with a parallel phenomenon of shifting M1 microglia to M2 microglia, the detailed relationship requires further investigation.

**Table 2:** The MST kinases in CNS diseases.

	Author/year	Disease	MST kinase	Main finding
Tumor	Costa et al.[99] /2012	Medulloblastoma	STK25	STK25 prevents medulloblastoma cells death via attenuating TrkA—STK25 signaling pathway
	Chao et al. [97] /2015	Glioblastoma	MST1	Mst1 prevents glioblastoma growth via attenuating AKT—mTOR signaling pathway
	Zhu et al.[91] /2015	Glioblastoma	MST1/2	miR-130b promotes glioblastoma growth via attenuating MST1/2—Lats—YAP/TAZ signaling pathway
	Zhang et al.[88] /2016	Glioblastoma	MST1/2	YAP/TAZ—BIRC5 signaling pathway induced by Lats down-regulation promotes glioblastoma growth
Vascular diseases	Voss et al.[106] /2007	CCM	STK25	Interaction between CCM2, CCM3 and STK25 mediates vascular development and CCM pathogenesis
	Zheng et al.[104] /2010	CCM	STK24/25	Interaction between CCMs and STKs mediates vascular development and CCM pathogenesis
	Zhao et al.[108] /2016	Cerebral ischemia	MST1	MST1 promotes cerebral-ischemia-induced microglia activation via Src—MST1—IκBα signaling pathway
	Weng et al.[112] /2016	Cerebral ischemia	MST1	Malibatol A prevents cerebral-ischemia-induced microglia activation via c-Abl—MST1 signaling pathway
	Yang et al.[115] /2016	VD	MST1	TSL protects neurons activity in VD via attenuating inflammatory reaction mediated by MST1—FOXO3 signaling pathway
Neurodegenerative diseases	Matsuki et al.[23] /2012	AD	STK25	Stk25 attenuating AD development via preventing Tau phosphorylation induced by Dab1 deficiency
	Lee et al.[130] /2013	ALS	MST1	MST1 mediates ALS development via interaction with SOD1
	Pan et al.[138]/2014	Prion diseases	MST1	c-Abl—MST1 signaling pathway promotes prion-induced neuralapoptosisin Prion diseases
Other CNS diseases	Zhang et al.[142]/2015	Spine injury	MST3b	Mst3b promotes neural regeneration in injured spinal cord
	Imitola et al.[13]/2015	2q37 microdeletion syndrome	STK25	STK25 deletion was the most interacting gene in neural development disorder of 2q37 microdeletion syndrome

The MST-related CNS diseases in four parts: tumor, vascular, neurodegenerative diseases and other CNS diseases. The study of multiple MST signaling pathways might provide us better therapeutic targets for the treatment of CNS diseases.

MST1 is also involved in the development of vascular dementia (VD) through the MST1-FOXO3 signaling pathway [115]. VD is a common age-related neural disease, usually a result of cerebrovascular disease, such as ischemic or hemorrhagic stroke [116]. The inflammation cascade is triggered by these cerebrovascular events, and the secondary inflammatory response initiates blood-brain barrier dysfunction, cerebral edema, and neuronal cell death [117-119]. These alterations play an important role in pathological development of VD [120, 121]. As previous discussed, the MST1-FOXO3 signaling pathway mediates oxidative stress-induced neuronal cell death [67]. Intresetingly, a recent study presented how Tanshinol (TSL),a traditional Chinese medicine for vascular disease, can protect the hippocampus and prevent learning and memory impairment via attenuatingMST1-FOXO3 signaling meditaed inflammatory process in a rat model of VD [115].

## Neurodegenerative diseases

MST kinases are involved in neurodegenerative diseases including Alzheimer’s disease (AD) and Amyotrophic lateral sclerosis (ALS). AD is the most common neurodegenerative disease. However, the mechanisms behind AD remain unclear [122, 123]. Excessive binding protein Tau phosphorylation is wildly accepted as a feature of AD, Parkinson’s, and Frontal Temporal Lobe Dementia [124, 125]. Docking protein Dab1 deficiency in Reelin signaling pathway can lead to Tau hyperphosphorylation. However, the physiological function of STK25 (performs neuronal polarization regulation and Golgi morphology) was found to have the ability to defend against the Dab1 effect in development of AD in rat model, demonstrating the anti-AD effect of STK25 [23]. Thus, the investigation of STK25 in AD is a topic of interest in AD treatment.

ALS is an adult neurodegenerative disease featured by the progressive loss of motor neurons in the motor cortex, brainstem, and spinal cord [126, 127]. Mutants of human superoxide dismutase 1 (SOD1) gene, which usually causes oxidative damage and apoptosis in motor neurons, and are the most acceptable mechanism of ALS pathogenesis [128, 129]. The recent study found that genetic ablation of MST1 can delay ALS onset and improve prognosis in human SOD1(G93A) mutant mice [130]. As previously mentioned, the redox protein thioredoxin-1 (Trx1) inhibits MST1 homodimerization and autophosphorylation after oxidative induction [131]. SOD1(G93A) reversed this process and promoted MST1 activation. Furthermore, SOD1(G93A) induced activation of p38 MAPK and caspases and abnormalities in autophagic flux formation, thereby contributing to the pathogenesis of ALS [130, 132-134].

Other MST kinases related neurodegenerative disorders are Prion diseases, characterized by the accumulation of a disease-associated abnormal prion protein and neuron apoptosis [135]. Evidence demonstrated oxidative induction is the main contributor to Prion diseases pathogenesis. This was proved by the PrP106-126 or PrP^Sc^ model, a common model system to study prion-induced neurodegeneration [136, 137]. PrP106-126 activates c-Abl and subsequently upregulates MST1 and pro-apoptotic gene BIM, leading to neuron apoptosis via mitochondrial dysfunction [138].

## Other CNS diseases

Spinal cord injury is a devastating neurological diseases associated with high morbidity and functional impairments impacting quality of life [139-141]. MST3b is activated by neurotrophic factors and promotes axon outgrowth in damaged adult optic nerve and radial nerve [14, 72]. Moreover, it was found that Mst3b also plays an important role in injured spinal cord neurons. Increased MST3b levels can facilitate axonal regeneration of spinal cord neurons in vivo and in vitro. MST3b interacts with small G protein Ras and MAPK kinase, promoting down-stream signaling pathways, including P42/44^MAPK^ and LIMK/Cofilin signaling pathway, which further modulates actin cytoskeleton, resulting in axon regeneration in spinal cord neurons [142].

The 2q37 microdeletion syndrome is a syndrome characterized by mild-moderate developmental or intellectual disability, abnormal short bones in the fingers and hands, obesity, special facial appearance, autism and lot CNS disorders, including seizures and hydrocephalus [143]. The loss of approximately 100 genes loss in the 2q37 band is the main cause of this syndrome. However, the definite phenotype in this type of gene deletion is still unknown [144]. A recent study suggested STK25 deletion was the important gene deletion contributing to the neural developmental disorders associated with this syndrome. It broadens our understanding on the causative genomic region of 2q37 microdeletion syndrome and gives us a specific gene regarding the development of the human cortex and corpus callosum [13] (Table 2).

## Conclusion

MST kinases are an essential part the signaling transduction pathways, maintaining numerous biological functions, such as controlling cell growth, cell migration, cell polarity and cell apoptosis. The regulation of MST kinases is complicated and current studies on the regulatory mechanisms are not well understood. Accumulating evidence suggests MST kinases have essential roles in different body systems. They are also important to the CNS when it comes to biological function and development of disease. Increasing investigation and understanding of MST signaling pathways including regulatory and pathological processes of MST kinases and their role in the CNS could provide the research and medical community with new therapeutic targets for human diseases.
